# Cross-border spread of a mosaic resistance (OXA-48) and virulence (aerobactin) plasmid in *Klebsiella pneumoniae*: a European Antimicrobial Resistance Genes Surveillance Network investigation, Europe, February 2019 to October 2024

**DOI:** 10.2807/1560-7917.ES.2025.30.27.2500439

**Published:** 2025-07-10

**Authors:** Marius Linkevicius, Erik Alm, Louise Roer, Olov Svartström, Maria Dada-Olorunwa, Kati Räisänen, Felix Reichert, Sophie Möller, Christina Clarke, Martin Cormican, Baiba Niedre-Otomere, Reinis Vangravs, Paulius Greičius, Olga Burduniuc, Maria Anton, Antoni P.A. Hendrickx, Sandra Witteveen, Vilhelm Müller, Daniel Palm, Diamantis Plachouras, Dominique L. Monnet, Henrik Hasman, Anke Kohlenberg

**Affiliations:** 1 European Centre for Disease Prevention and Control, Stockholm, Sweden; 2 National Reference Laboratory for Antimicrobial Resistance, Department of Bacteria, Parasites and Fungi, Statens Serum Institut, Copenhagen, Denmark; 3 European Union Reference Laboratory for Public Health in the Field of Antimicrobial Resistance (AMR) in Bacteria (EURL-PH-AMR); 4 European Society for Clinical Microbiology and Infectious Diseases (ESCMID) Study Group for Mobile Elements and Plasmids (ESGMAP), Basel, Switzerland; 5 Finnish Institute for Health and Welfare, Helsinki, Finland; 6 Department of Infectious Disease Epidemiology, Robert Koch Institute, Berlin, Germany; 7 German National Reference Centre for Multidrug-resistant Gram-negative Bacteria, Department of Medical Microbiology, Ruhr-University Bochum, Bochum, Germany; 8 Galway Reference Laboratory Service, Galway University Hospital, Galway, Ireland; 9 School of Medicine, University of Galway, Galway, Ireland; 10 National Microbiology Reference Laboratory of Latvia, Laboratory “Latvian Centre of Infectious Diseases”, Laboratory Service, Riga East University Hospital, Riga, Latvia; 11 National Public Health Surveillance Laboratory, Vilnius, Lithuania; 12 National Agency for Public Health, Ministry of Health, Chisinau, Moldova; 13 Centre for Infectious Disease Control, National Institute for Public Health and the Environment (RIVM), Bilthoven, the Netherlands; 14 Public Health Agency of Sweden, Solna, Sweden

**Keywords:** Carbapenemase-producing Enterobacterales, surveillance, whole genome sequencing, cross-border spread, antimicrobial resistance, virulence

## Abstract

An investigation of the European Antimicrobial Resistance Genes Surveillance Network (EURGen-Net) detected the same mosaic IncHI1B(pNDM-MAR) resistance (OXA-48) and virulence (aerobactin) plasmid in 492 *Klebsiella pneumoniae *isolates from eight European Union countries during 2019–2024. Involvement of various *K. pneumoniae* sequence types (STs), multiple introductions, followed by large clonal outbreaks of three STs (ST147, ST392, ST45) carrying the plasmid in two countries indicate a high risk for further spread of this plasmid and a potential for difficult-to-treat *K. pneumoniae* infections to rise.

Starting in 2022, Latvia and Lithuania experienced outbreaks of *bla*_OXA-48_-carrying *Klebsiella pneumoniae* in their healthcare systems. Different *K. pneumoniae* sequence types (STs) were involved in both countries, mainly ST147 in Latvia, and ST392 (part of clonal group CG147), ST395 and ST45 in Lithuania [1]. Long-read sequencing of one ST392 isolate in Lithuania and three ST147 isolates in Latvia identified an IncHI1B(pNDM-MAR) mosaic plasmid carrying both resistance (*bla*_OXA-48_) and virulence genes (aerobactin operon; *iucABCD*, *iutA*). To determine the extent of this plasmid’s spread, the European Centre for Disease Prevention and Control (ECDC) initiated a cross-border outbreak investigation supported by the European Union (EU) reference laboratory for public health in the field of antimicrobial resistance in bacteria (EURL-PH-AMR).

## Data collection and analysis

**Figure 1:**
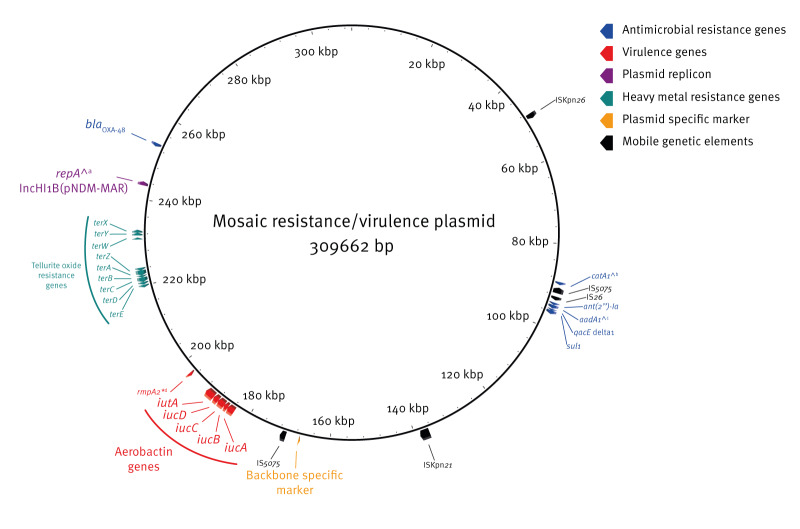
Diagram of a mosaic resistance and virulence plasmid representative sequence with the key markers *bla*_OXA-48_, aerobactin genes (*iucABCD*, *iutA*) and IncHI1B(pNDM-MAR) replicon as well as a backbone specific oligonucleotide marker

On 17 July 2024, via its European surveillance portal for infectious diseases (EpiPulse), ECDC requested national reference laboratories that participate in the European Antimicrobial Resistance Genes Surveillance Network (EURGen-Net) to report isolates, which, based on short-read sequencing results, had key markers of the plasmid, namely *bla*_OXA-48_, aerobactin genes (*iucABCD*, *iutA*), and IncHI1B(pNDM-MAR) replicon (Figure 1). Eight countries replied and submitted sequences of 685 *K. pneumoniae* isolates fulfilling these inclusion criteria together with epidemiological and microbiological data provided according to a standard template [2]. Moldova also contributed sequences of 29 *K. pneumoniae* isolates. In addition, we completed the dataset with sequences of *K. pneumoniae* isolates exhibiting the key markers from previous ECDC investigations (n = 76) and from the National Center for Biotechnology Information (NCBI) Pathogen Detection and NCBI RefSeq databases (n = 1,139), as further detailed in the Supplement, excluding duplicates. A single genome sequence of an *Escherichia coli* isolate with the key markers was submitted by Latvia.

IS: insertion sequence.

The key markers are annotated in bold font. Mutations identified when comparing to the reference sequences of Kleborate or PlasmidFinder tools are detailed below.

^a^ *repA*^: silent G42A and T243A substitutions.

^b^ *catA1*^: silent C585T substitution.

^c^ *aadA1*^: silent C750T substitution.

^d^ rmpA2*: silent T114C substitution; insertion of eight guanosines at position 286 leading to a frameshift and truncated amino acid sequence.

Short-read sequences were assembled using SPAdes v3.14.1 or later versions [3] and long- reads using Flye v2.9.4 [4]. Alleles were called with ChewBBACA v3.3.10 [5] using the *K. pneumoniae sensu lato* core genome multilocus sequence typing (cgMLST) scheme [6]. Of 1,929 genomes, 1,912 passed quality control (≥ 90% alleles called with genome size of 4.87−6.5 Mbp). The STs (Institute Pasteur scheme), antibiotic resistance and virulence genes were determined using Kleborate v3.1.3 [7] and plasmid replicons using PlasmidFinder v2.0.1 (database from 1 March 2023) [8]. Mapping of resistance and virulence genes, plasmid replicon and mobile genetic elements was performed using MobileElementFinder v1.0.3 (database from 9 June 2020) [9] and visualised with basic local alignment search tool (BLAST) Ring Image Generator (BRIG) v0.95 [10].

The key markers, with in addition, a plasmid-specific oligonucleotide, as described in the Supplement, were used to identify the mosaic plasmid in the short-read dataset. Based on these, 604 of 1,912 *K. pneumoniae* genomes were found to carry the plasmid including 492 genomes submitted to ECDC (Table) and 112 from the NCBI databases. Of these, additional long-read or plasmid-only sequences were obtained for 45 and 18 *K. pneumoniae* isolates, respectively. The submitted long-read genome of the *E. coli* isolate was also positive for the mosaic plasmid.

**Table:** Distribution of the mosaic resistance and virulence plasmid-carrying *Klebsiella pneumoniae* genomes submitted to ECDC, by ST and country, February 2019–October 2024 (n = 492 isolates)

Country^a^	Years of detection	Mosaic plasmid-carrying *K. pneumoniae* isolates		
		ST147	Other STs^b^	Total
Latvia	2022–2024	304	1	**305**
Lithuania	2023–2024	5	105	**110**
Germany	2022–2024	22	0	**22**
The Netherlands	2022–2024	18	0	**18**
Denmark	2022–2023	11	0	**11**
Sweden	2022–2024	11	0	**11**
Finland	2022–2024	8	0	**8**
Ireland	2019, 2022, 2024	6	1	**7**
**Total**	**2019, 2022–2024**	**385**	**107**	**492**

ECDC: European Centre of Disease Prevention and Control; ST: sequence type.

^a^ Countries are listed by decreasing number of isolates. Of 29 isolates from Moldova, 28 belonged to ST395 and one to ST23, but none carried the mosaic plasmid specific oligonucleotide marker.

^b^ Other STs included ST45 (n = 55), ST392 (n = 46), ST4110 (n = 2), ST15 (n = 1) and ST29 (n = 1) all in Lithuania, ST395 (n = 1) in Ireland, and ST8282 (n = 1) in Latvia.

The *K. pneumoniae* isolates carrying the mosaic plasmid from the NCBI databases (n = 112) were from Germany (n = 30), Russia (n = 25), Spain (n = 17), the Netherlands (n = 16), Sweden (n = 8), the United States (n = 7), Slovakia (n = 4), Ukraine (n = 4) and Israel (n = 1). These isolates were mainly *K. pneumoniae* ST147 (n = 106), followed by ST395 (n = 4), ST39 (n = 1) and ST8324 (n = 1).

## Genomic characteristics

**Figure 2:**
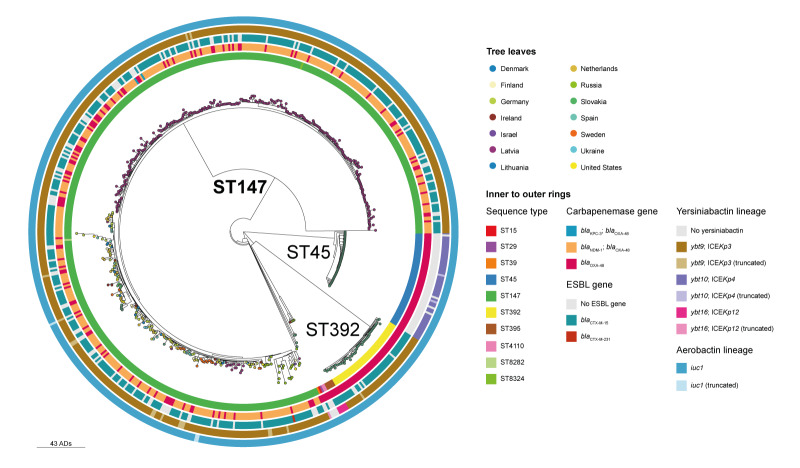
Phylogenetic tree of *Klebsiella pneumoniae* isolates carrying the mosaic resistance and virulence plasmid, February 2019–October 2024 (n = 604)

AD: allelic difference; ESBL: extended-spectrum β-lactamase; ST: sequence type.

Of 604 *K. pneumoniae* isolates identified with the mosaic plasmid, 380 (most ST147; n = 378) carried an additional carbapenemase gene, either *bla*_NDM-1_ (n = 378) or *bla*_KPC-3_ (n = 2) (Figure 2). More than 70% of the 604 isolates (n = 428), mainly of ST147 (379/428; 89%) and of ST392 (41/428; 10%) both belonging to the same clonal group CG147, also carried extended-spectrum β-lactamase (ESBL) genes, either *bla*_CTX-M-15_ (n = 427) or *bla*_CTX-M-231_ (n = 1). Long-read assemblies of 17 ST147 isolates carrying *bla*_NDM-1_ and *bla*_CTX-M-15_ showed that in most cases (n = 16) these genes were co-located on an IncFIB(pQil) plasmid and not the mosaic IncHI1B(pNDM-MAR) plasmid. In the remaining case, the *bla*_CTX-M-15_ gene was on an IncFIB(pQil) plasmid, while *bla*_NDM-1_ gene was in another genomic location. In contrast, other genes known to confer resistance to aminoglycosides, sulfamethoxazole, chloramphenicol, quaternary ammonium compounds, and heavy metals (*ter* genes) were detected on the mosaic plasmid (Figure 1).

Mutations of genes encoding outer membrane porins (OMPs) were detected in 524 (87%) of 604 isolates, of which both *ompK35* and *ompK36* were mutated in most cases (n = 489; 93%).

Most (n = 596; 99%) isolates had a Kleborate virulence score of four indicating presence of both yersiniabactin and aerobactin. Different yersiniabactin lineages were detected in different STs (Figure 2). All isolates harboured the *iuc1* aerobactin lineage present on the mosaic plasmid. Of 604 isolates, 171 had other virulence genes, *rmpA2* (n = 166; 28%) or *rmpA* (n = 5; 1%). Of those, 167 carried truncated RmpA2 (n = 162) or RmpA (n = 5), and four isolates an intact RmpA2. Available *K. pneumoniae* long-read assemblies (n = 45) and mosaic plasmid-only contigs (n = 18) showed the location of *rmpA2* on the mosaic plasmid (Figure 1).

Genomic relatedness within STs was determined with single linkage cut-off of 10 allelic differences detecting three large clusters with more than five isolates in ST147 (n = 476, including two single locus variants ST8282 and ST8324), ST45 (n = 54) and ST392 (n = 46).

## Epidemiological characteristics

**Figure 3:**
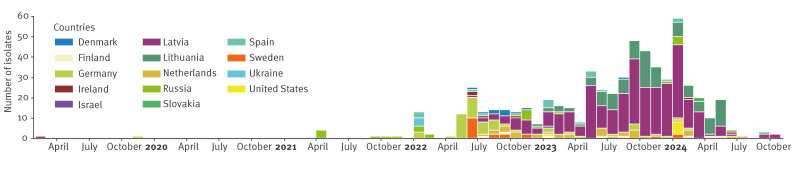
Time distribution^a^ of *Klebsiella pneumoniae* isolates carrying the mosaic resistance and virulence plasmid by country, February 2019–October 2024 (n = 604)

^a^ The time distribution above should not be interpreted as an epidemic curve as it may be affected by bias in detection and reporting and does not reflect true temporal trends in incidence. Of note, data for this study were collected between 17 July 2024 and 1 October 2024 and isolates with later sampling dates were not included.

The earliest *K. pneumoniae* isolate with the mosaic plasmid (belonging to ST395) was detected in February 2019 in Ireland, followed by another ST395 isolate from Germany with a link to Russia [11] in November 2019 (Figure 3). The first *K. pneumoniae* ST147 isolates with the mosaic plasmid were detected in Russia in 2021, followed, from 2022 onwards, by detections in 13 other countries, including 10 EU countries. From 2023 onwards, *K. pneumoniae* isolates of other STs carrying the plasmid were detected in Lithuania.

The following epidemiological characteristics were assessed for the 492 isolates for which sequences were submitted to ECDC with standardised metadata. Of 376 isolates with associated patient information on sex, 239 (64%) were males and 137 (36%) females. For 381 isolates with associated patient information on age, the median age was 66 years (range: 2–94 years). Of 444 isolates with information on the type of sample, urine was most frequent (n = 142; 32%), followed by lower respiratory tract (n = 58; 13%), blood (n = 50; 11%) and skin and soft tissue samples (n = 42; 9%) while 152 (34%) isolates originated from various other sample types including screening samples. Information on a reported epidemiological link including either prior hospitalisation in or prior travel to another country was available for 50 *K. pneumoniae* ST147 isolates from six countries (Denmark, Germany, Finland, Latvia, the Netherlands, and Sweden) and 49 of these had a link to Ukraine and one to Poland.

## Discussion

The IncHI1B(pNDM-MAR) replicon has been previously described in a study published in 2012 regarding a pNDM-MAR plasmid extracted from a *K. pneumoniae* ST15 isolate collected in Morocco [12]. Since, many findings of IncHI1B(pNDM-MAR) plasmids have been reported worldwide [13]. Here, we focused our investigation on one specific IncHI1B(pNDM-MAR) plasmid with converged resistance (e.g. *bla*_OXA-48_) and virulence (e.g. aerobactin operon *iucABCD*, *iutA*) genes. We detected this IncHI1B(pNDM-MAR) mosaic resistance−virulence plasmid in eight EU countries, which submitted data to ECDC and in six other countries through public data, confirming cross-border spread.

The earliest detection of the mosaic resistance−virulence plasmid was in 2019 in *K. pneumoniae* ST395 consistent with previous descriptions of mosaic plasmids in this ST [14]. However, detection of this plasmid in participating EU countries mainly occurred from 2022 onwards. The timing and the identified epidemiological links suggest multiple introductions, mainly of *K. pneumoniae* ST147, through medical transfers from Ukraine followed by clonal spread resulting in a large outbreak in Latvia and plasmid transfer. The plasmid was also detected in seven other *K. pneumoniae* STs and one *E. coli* isolate indicating its capacity to transfer across *K. pneumoniae* STs and potentially other Enterobacterales species. Subsequently, *K. pneumoniae* ST45 and ST392, both carrying the plasmid, caused outbreaks in Lithuania [1].

The rapid global spread of OXA-48-like carbapenemases presents a serious threat for healthcare systems [15]. Moreover, the plasmid-mediated acquisition of aerobactin genes by *K. pneumoniae* may enhance virulence potentially resulting in an increase of infections in the vulnerable patients in European healthcare settings. Although clinical outcome data were not available for this investigation, even a slight rise in virulence might elevate the number or severity of *K. pneumoniae* infections in Europe. The estimated incidence of *K. pneumoniae* bloodstream infections is increasing in the EU, although improved blood culture sampling and better coverage of surveillance may also contribute to this increase [16].

The high-risk ST147 lineage is known for its capacity to spread in healthcare settings. In this investigation, the ST147 isolates were carrying chromosomal yersiniabactin, with virulence potentially further amplified by aerobactin acquisition. While aerobactin is an important marker of hypervirulence in *K. pneumoniae* [17], it alone is insufficient to confer a hypervirulent phenotype [18]. Other hypervirulence markers, such as *rmpA* and *rmpA2* [19], were detected on the mosaic plasmid, but in these isolates RmpA was truncated and only functional RmpA was shown to increase virulence [18]. Additionally, the *ter* operon was linked to enhanced fitness during urinary tract infections and gut colonisation [20].

This investigation has some limitations including incomplete epidemiological data and absence of clinical outcome data. In addition, only eight EU countries responded to the ECDC’s EpiPulse request. The spread of the mosaic plasmid described in this investigation may remain undetected due to a lack of genomic surveillance for virulence genes and plasmids. While this investigation highlights one example, similar mosaic plasmids have been reported [21], but the extent of their spread in the EU countries is currently unclear.

## Conclusion

The multiple introductions of the mosaic plasmid across EU countries, the involvement of different *K. pneumoniae* STs and the large outbreaks associated with three STs highlight a high risk of further spread of the mosaic plasmid including expansion of STs carrying this plasmid. Enhancement of laboratory capacity for detection and characterisation of high-risk lineages and related plasmids, surveillance programmes and enhanced infection prevention and control measures are therefore urgently required.

## Data Availability

The national whole genome sequencing data of isolates with the mosaic plasmid were deposited in the European Nucleotide Archive under accession numbers PRJEB35890, PRJEB60743, PRJEB74083, PRJEB75178, PRJEB89771, PRJEB89810, PRJNA1076808, PRJNA1106484, PRJNA1143178, PRJNA288601, PRJNA648389, PRJNA657553, PRJNA903550, PRJEB89896, PRJEB76821, and PRJNA1268013. More information can be found in the Supplementary Table.

## Supplementary Data

228000 Supplement

## Supplementary Data

15000 Supplementary Table
