# Authors’ correction for Euro Surveill. 2025;30(27)

**DOI:** 10.2807/1560-7917.ES.2025.30.47.251127c

**Published:** 2025-11-27

**Authors:** Eurosurveillance editorial team

**Affiliations:** 1 European Centre for Disease Prevention and Control (ECDC), Stockholm, Sweden

In the article entitled ‘Cross-border spread of a mosaic resistance (OXA-48) and virulence (aerobactin) plasmid in *Klebsiella pneumoniae*: a European Antimicrobial Resistance Genes Surveillance Network investigation, Europe, February 2019 to October 2024’ by Linkevicius et al., published on 10 July 2025, the sequence type (ST) 8282 of *Klebsiella pneumoniae* was corrected to ST8278 in the main text, the legend of Figure 2, the footnote ‘b’ of the Table, as well as inside the Supplementary Table. These corrections were implemented on 21 November 2025 at the author‘s request.

