# Supplementary material for: Cross-border spread of a mosaic resistance (OXA-48) and virulence (aerobactin) plasmid in Klebsiella pneumoniae: a European Antimicrobial Resistance Genes Surveillance Network investigation, Europe, February 2019 to October 2024
Source: Euro Surveill. 2025 Jul 10;30(27):2500439. doi: 10.2807/1560-7917.ES.2025.30.27.2500439 (PMC12262110; doi:10.2807/1560-7917.ES.2025.30.27.2500439)
Supplement: Supplement [file 25-00439_LINKEVICIUS_Supplement.pdf]

Cross-border spread of a mosaic resistance (OXA-48) and virulence (aerobactin) plasmid in *Klebsiella pneumoniae*: a European Antimicrobial Resistance Genes Surveillance Network investigation, Europe, February 2019 to October 2024

This supplementary material is hosted by Eurosurveillance as supporting information alongside the article 'Cross-border spread of a mosaic resistance (OXA-48) and virulence (aerobactin) plasmid in *Klebsiella pneumoniae*: a European Antimicrobial Resistance Genes Surveillance Network investigation, Europe, February 2019 to October 2024', on behalf of the authors, who remain responsible for the accuracy and appropriateness of the content. The same standards for ethics, copyright, attributions and permissions as for the article apply. Supplements are not edited by Eurosurveillance and the journal is not responsible for the maintenance of any links or email addresses provided therein.

Generation of plasmid backbone specific oligonucleotide marker

Public domain data for this investigation were collected from National Center for Biotechnology Information (NCBI) Pathogen Detection (accessed on 5 February 2025) and NCBI RefSeq (accessed on 22 March 2025) databases. Complete plasmid sequences were assembled from PacBio long reads generated from two representative *Klebsiella pneumoniae* ST147 isolates using Flye v2.9.4 [1]. The resulting 310 kbp plasmid contigs (GenBank accessions PV691627 and PV754604), containing all the key markers, were close to identical in both isolates. K-mer elimination by cross-reference (KEC) software [2] in exclusion mode was used to identify candidate oligonucleotide markers for the mosaic plasmid using 93 closely related but not identical reference sequences downloaded from the NCBI core_nt database as non-target sequences. One oligonucleotide marker (ATTCCGAACATAAATGCAATGATGAGCAGTAAGAGCACGCCCATTTGCAGCGCCGGAAG) was identified, and verified against available *K. pneumoniae* long-read assemblies from Latvia and Lithuania (n=45), plasmid-only sequences (n=18) from the Netherlands and all available complete IncHI1B(pNDM-MAR) plasmid sequences in the NCBI RefSeq database [3] (accessed on 22 March 2025 with a total of 114793 *K. pneumoniae* sequences) as well as the plasmid database [4] (PLSDB; accessed on 2 February 2025 with a total of 72556 closed plasmid sequences) indicating high specificity. Exact match (100% identity and 100% coverage) against this oligonucleotide marker as well as the other three plasmid-specific key markers (presence of $bla_{OXA\text{-}48}$, *iucABCD+iutA* and IncHI1B(pNDM-MAR)) was used as a proxy for presence of the mosaic plasmid when screening the short-read and long-read datasets.

References

1. Kolmogorov M, Yuan J, Lin Y, Pevzner PA. Assembly of long, error-prone reads using repeat graphs. Nat Biotechnol. 2019 May;37(5):540-6. https://doi.org/10.1038/s41587-019-0072-8 PMID: 30936562

2. Beran P, Stehlíková D, Cohen SP, Čurn V. KEC: unique sequence search by K-mer exclusion. Bioinformatics. 2021 Oct 11;37(19):3349-50. https://doi.org/10.1093/bioinformatics/btab196 PMID: 33755102

3. O'Leary NA, Wright MW, Brister JR, Ciufo S, Haddad D, McVeigh R, et al. Reference sequence (RefSeq) database at NCBI: current status, taxonomic expansion, and functional annotation. Nucleic Acids Res. 2016 Jan 4;44(D1):D733-45. https://doi.org/10.1093/nar/gkv1189 PMID: 26553804

4. Schmartz GP, Hartung A, Hirsch P, Kern F, Fehlmann T, Müller R, et al. PLSDB: advancing a comprehensive database of bacterial plasmids. Nucleic Acids Res. 2022 Jan 7;50(D1):D273-d8. https://doi.org/10.1093/nar/gkab1111 PMID: 34850116
